# Supplementary material for: Pectin Nanoparticle-Loaded Soft Coral Nephthea sp. Extract as In Situ Gel Enhances Chronic Wound Healing: In Vitro, In Vivo, and In Silico Studies
Source: Pharmaceuticals (Basel). 2023 Jul 3;16(7):957. doi: 10.3390/ph16070957 (PMC10383585; doi:10.3390/ph16070957)
Supplement: Supplementary file 1 [file pharmaceuticals-16-00957-s001.zip › pharmaceuticals-2445750-supplementary.pdf]

Supplementary Materials

Pectin Nanoparticle-Loaded Soft Coral *Nephthea* sp. Extract as *In Situ* Gel Enhances Chronic Wound Healing: *In Vitro, In Vivo,* and *In Silico* Studies

Nevine H. Hassan [1], Seham S. El-Hawary [2], Mahmoud Emam [3], Mohamed A. Rabeh [4], Mohamed A. Tantawy [5,6,7], Mohamed Seif [8], Radwa M. A. Abd-Elal [9,*], Gerhard Bringmann [10], Usama Ramadan Abdelmohsen [11,12,*] and Nabil M. Selim [2,*]

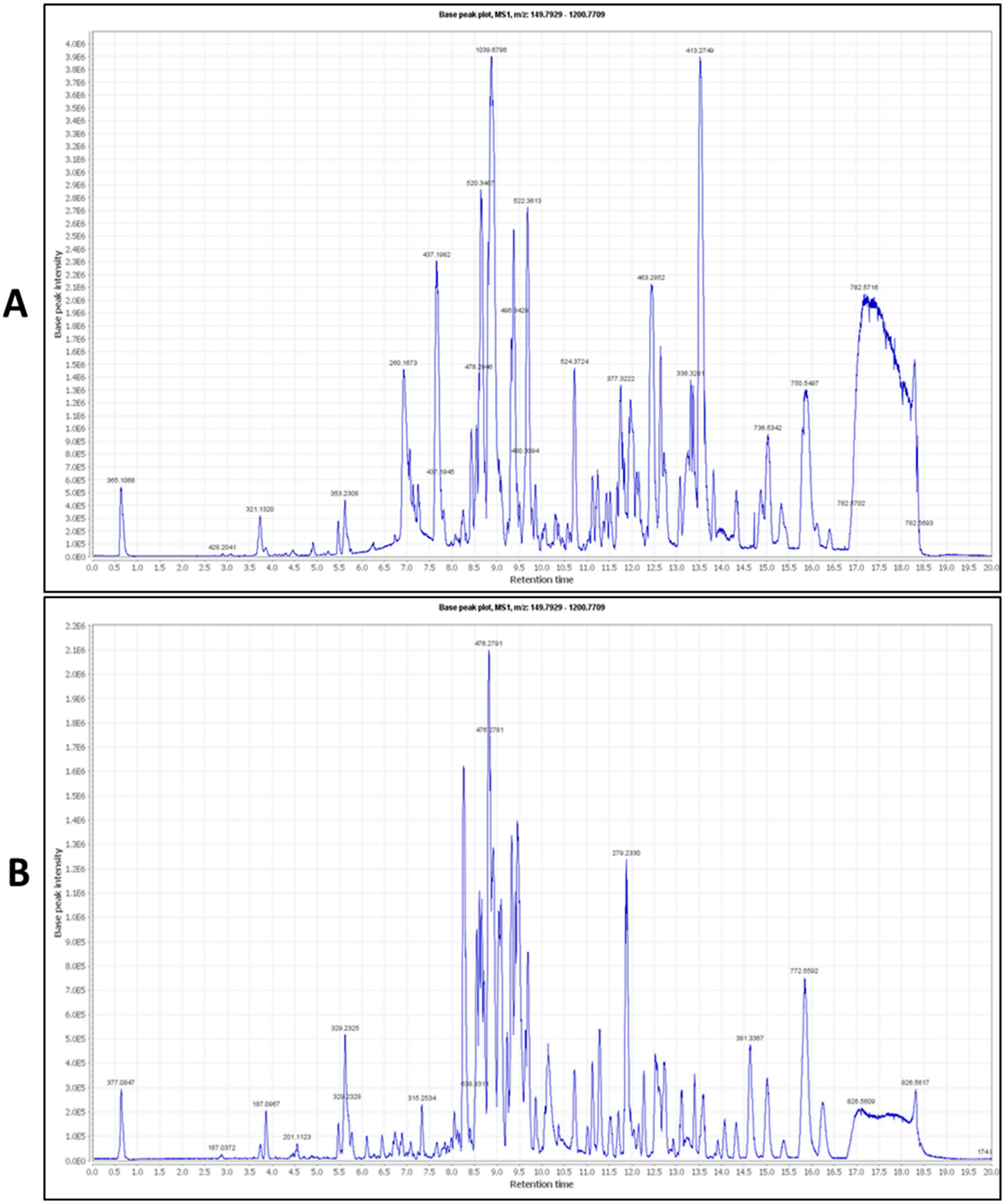

Figure S1. Total ion chromatogram of *Nephthea* sp. extract recorded in (A) positive ionization mode and (B) negative ionization mode.

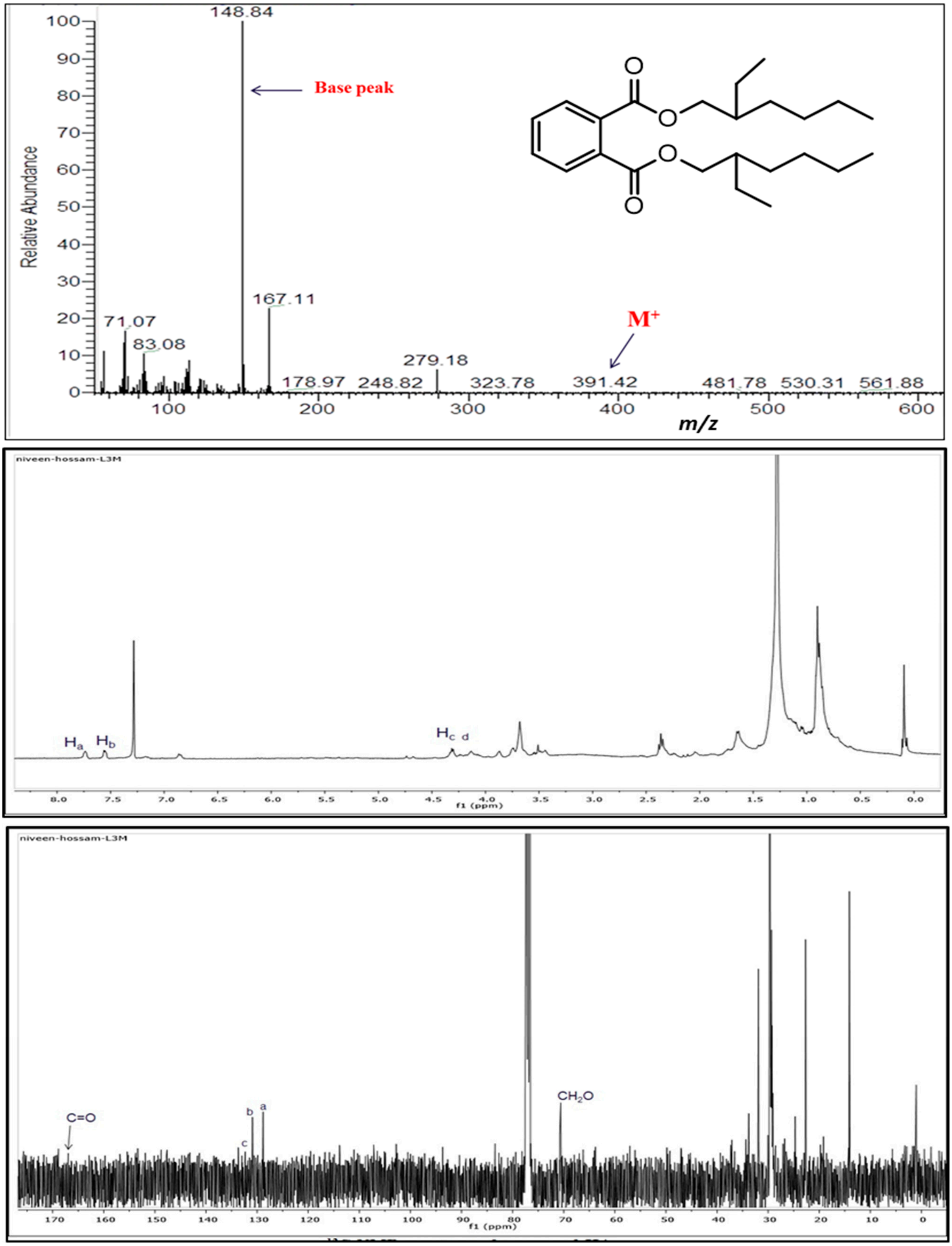

Figure S2. ESI-MS as well as ^{1}H NMR and ^{13}C NMR spectra of compound **12** (bis-(2-ethylhexyl)-phthalate) measured in $CDCl_3$

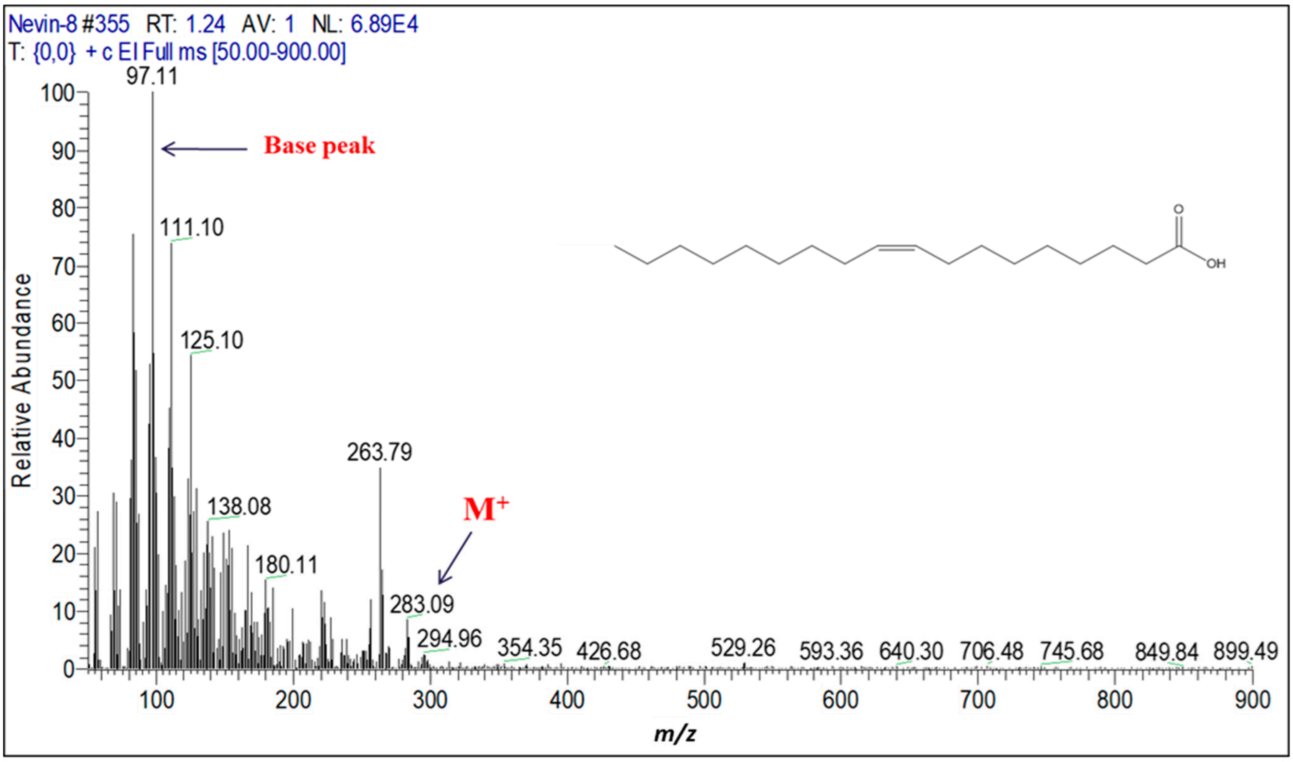

Figure S3. ESI-MS spectra of compound **18** (oleic acid) measured in $CDCl_3$

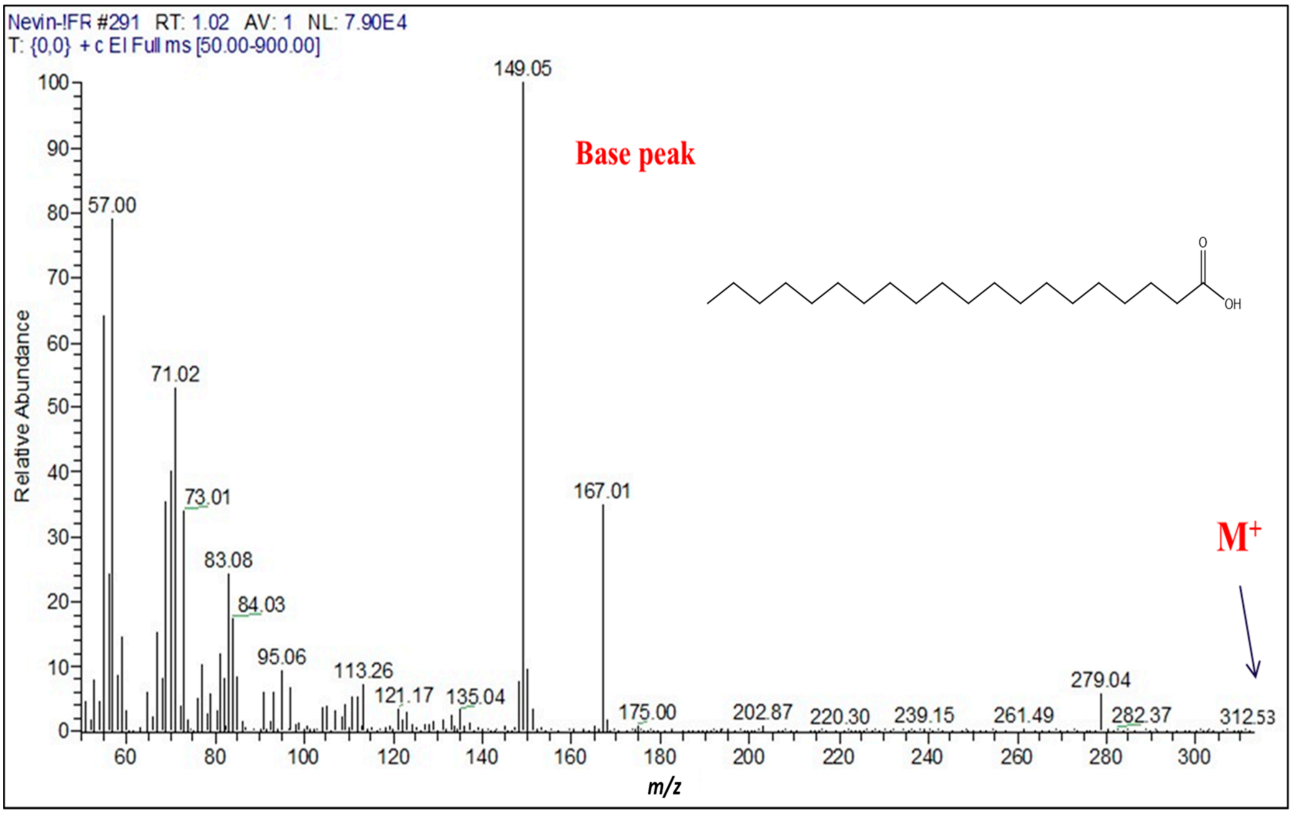

Figure S4. ESI-MS spectra of compound **20** (arachidic acid) measured in $CDCl_3$

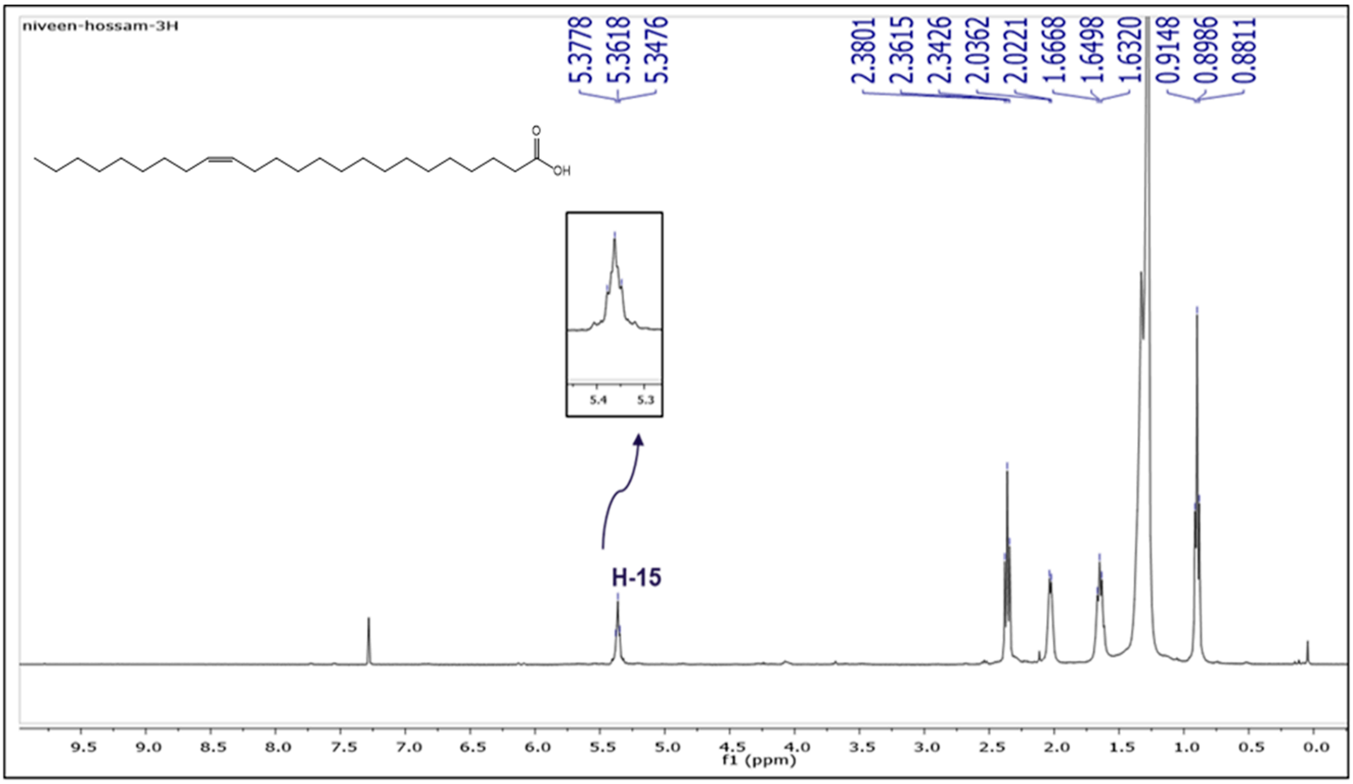

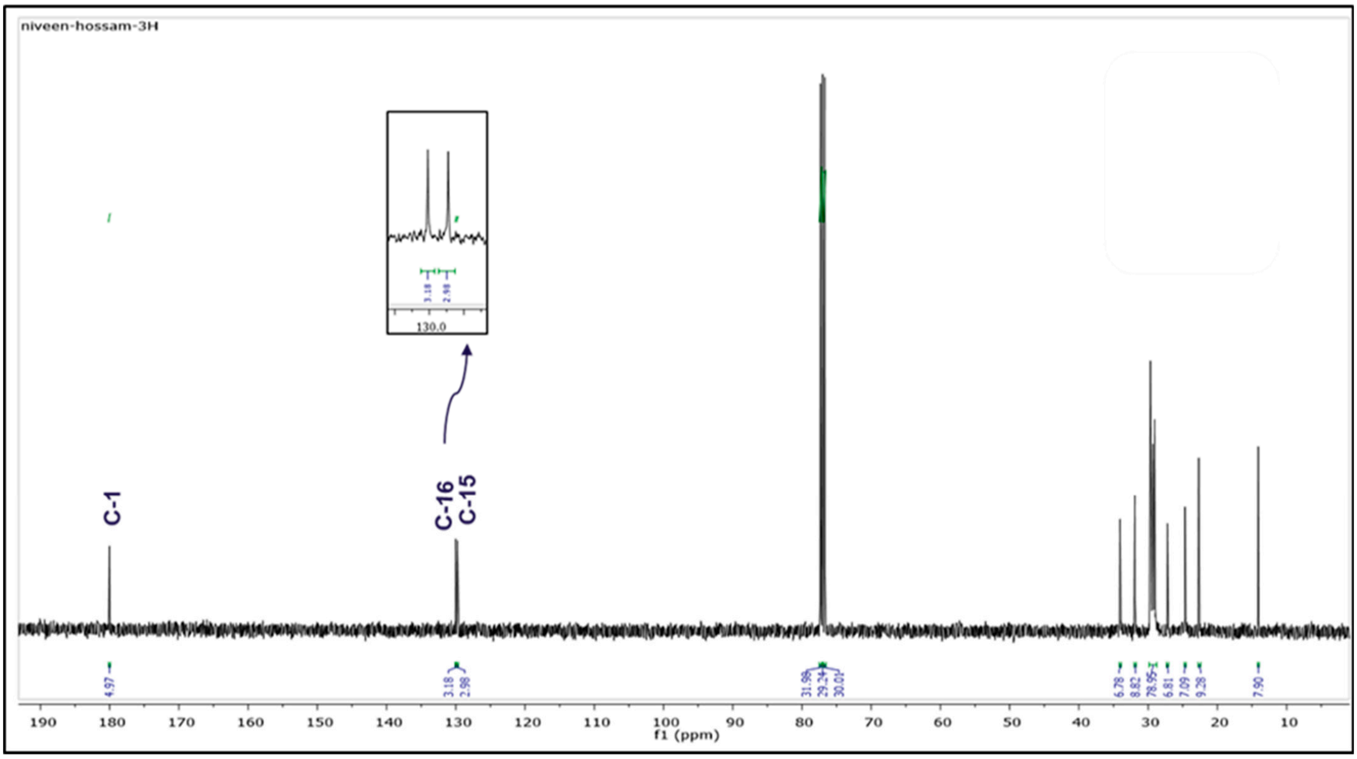

Figure S5. ^{1}H NMR and ^{13}C NMR spectra of compound **24** (nervonic acid) measured in $CDCl_3$

Table S1. Medicinal- chemistry properties for the most active compounds.

Parameter	Compound		Comment
	Chabrolohydroxybenzoquinone F (**13**)	Isogosterone B (**9**)	
QED	0.245	0.416	A measure of drug-likeness based on the concept of desirability; attractive: > 0.67; unattractive: 0.49~0.67; too complex: < 0.34
Synth	4.146	5.755	The synthetic accessibility score is designed to estimate the ease of synthesis of drug-like molecules. SA score > 6: difficult to synthesize; SA score < 6: easy to synthesize
Fsp^3	0.481	0.759	The number of sp^3 hybridized carbons / total carbon count, correlating with melting point and solubility. $Fsp^3 \geq 0.42$ is considered a suitable value
MCE-18	28	114.529	MCE-18 stands for medicinal chemistry evolution. MCE-18 ≥ 45 is considered a suitable value
Pfizer	Accepted	Accepted	Log P > 3; TPSA < 75; compounds with a high log P (> 3) and low TPSA (< 75) are likely to be toxic

Table S2. Absorption profile for the most active compounds.

Parameter	Compound		Comment
	Chabrolohydroxybenzoquinone F (**13**)	Isogosterone B (**9**)	
HIA	0.617	0.035	Human intestinal absorption.<br>More close to 1 considered to be highly absorbed by intestine
Caco-2 permeability	-4.802	-4.656	Toxicity on normal cells, such as Caco-2 cells. Optimal: higher than -5.15 Log unit
MDCK permeability	3.23E-05	2.11E-05	Toxicity on normal cells such as MDCK cells;<br>low permeability: $< 2 \times 10^6$ cm/s<br>medium permeability: $2–20 \times 10^6$ cm/s<br>high passive permeability: $> 20 \times 10^6$ cm/s

Table S3. Distribution properties for the most active compounds.

Parameter	Compound		Comment
	Chabrolohydroxybenzoquinone F (**13**)	Isogosterone B (**9**)	
BBB	0.019	0.974	Blood-brain barrier (BBB) penetration<br>More close to 1 considered to be highly preamble by BBB
PPB	98.61%	86.57%	Plasma protein binding (PPB)<br>Optimal drugs should have a value < 90%.
VDss	0.476	0.931	Volume distribution (VD)<br>Optimal: 0.04-20 L/kg
Fu	1.08%	8.88%	The fraction unbound (Fu) in plasma;<br>low: < 5%; middle: 5~20%; high: > 20%

Table S4. Metabolism profile for the most active compounds.

Parameter	Compounds		Comment
	Chabrolohydroxybenzoquinone F (**13**)	Isogosterone B (**9**)	
CYP1A2-inh	0.267	0.003	Closer to 1: considered to be an inhibitor.
CYP1A2-sub	0.36	0.106	Closer to 1: considered to be a substrate.
CYP2C19-inh	0.449	0.024	Closer to 1: considered to be an inhibitor.
CYP2C19-sub	0.526	0.721	Closer to 1: considered to be a substrate.
CYP2C9-inh	0.595	0.09	Closer to 1: considered to be an inhibitor.
CYP2C9-sub	0.944	0.107	Closer to 1: considered to be a substrate.
CYP2D6-inh	0.905	0.005	Closer to 1: considered to be an inhibitor.
CYP2D6-sub	0.103	0.047	Closer to 1: considered to be a substrate.
CYP3A4-inh	0.809	0.732	Closer to 1: considered to be an inhibitor.
CYP3A4-sub	0.215	0.7	Closer to 1: considered to be a substrate.

Table S5. Excretion for the most active compounds.

Parameter	Compounds		Comment
	Chabrolohydroxybenzoquinone F (**13**)	Isogosterone B (**9**)	
CL	11.78	12.429	Clearance (CL): a compound that has a clearance > 15 mL/min/kg is considered to have a high clearance, while a compound that has a clearance from 5 to 15 mL/min/kg has a moderate clearance, and a compound that has a clearance < 5 mL/min/kg has a low clearance.
T12	0.484	0.485	Closer to 1: considered to have a long half-life.
